# RbCa_2_Nb_3_O_10_ from X-ray powder data

**DOI:** 10.1107/S1600536809018157

**Published:** 2009-05-23

**Authors:** Zhen-Hua Liang, Kai-Bin Tang, Qian-Wang Chen, Hua-Gui Zheng

**Affiliations:** a Department of Chemistry, University of Science and Technology of China, Hefei, Anhui 230026, People’s Republic of China; b Department of Nanomaterials and Nanochemistry, Hefei National Laboratory for Physical Sciences at Microscale, University of Science and Technology of China, Hefei, Anhui 230026, People’s Republic of China

## Abstract

Rubidium dicalcium triniobate(V), RbCa_2_Nb_3_O_10_, has been synthesized by solid-state reaction and its crystal structure refined from X-ray powder diffraction data using Rietveld analysis. The compound is a three-layer perovskite Dion–Jacobson phase with the perovskite-like slabs derived by termination of the three-dimensional CaNbO_3_ perovskite structure along the *ab* plane. The rubidium ions (4/*mmm* symmetry) are located in the inter­stitial space.

## Related literature

For the synthesis of RbCa_2_Nb_3_O_10_, see: Dion *et al.* (1981 ▶). For related three-layer Dion–Jacobson analogues, see: CsCa_2_Nb_3_O_10_ (Dion *et al.*, 1984 ▶); RbSr_2_Nb_3_O_10_ (Thangadurai *et al.*, 2001 ▶); KCa_2_Nb_3_O_10_ (Fukuoka *et al.*, 2000 ▶). For the application of Dion–Jacobson phases, see: Thangadurai *et al.* (2001 ▶); Li *et al.* (2007 ▶); Ida *et al.* (2008 ▶); Compton & Osterloh (2009 ▶). For properties of RbCa_2_Nb_3_O_10_, see: Thangadurai & Weppner (2001 ▶, 2004 ▶); Byeon *et al.* (2003 ▶).

## Experimental

### 

#### Crystal data

RbCa_2_Nb_3_O_10_
*M* *_r_* = 604.34
Tetragonal, 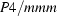

*a* = 3.85865 (6) Å
*c* = 14.9108 (3) Å
*V* = 222.01 (1) Å^3^
*Z* = 1
Cu *K*α radiation
*T* = 298 K
Specimen shape: flat sheet
10 × 15 × 1 mm
Specimen prepared at 1423 K
Particle morphology: plate-like, white

#### Data collection

PANalytical X’pert PRO diffractometer
Specimen mounting: packed powder pellet
Specimen mounted in reflection mode
Scan method: continuous
2θ_min_ = 10.0, 2θ_max_ = 110.0°
Increment in 2θ = 0.02°

#### Refinement

*R*_p_ = 0.035
*R*_wp_ = 0.053
*R*_exp_ = 0.008
*S* = 2.54
Wavelength of incident radiation: 1.54178 Å
Profile function: pseudo-Voigt
238 reflections
26 parameters
Preferred orientation correction: March–Dollase (Dollase, 1986 ▶) AXIS 1 Ratio = 0.95964, *h* = *k* = 0, *l* = 1; correction range: min = 0.94007, max = 1.13156

### 

Data collection: *X’pert Data Collector* (PANalytical, 2003 ▶); cell refinement: *GSAS* (Larson & Von Dreele, 2000 ▶) and *EXPGUI* (Toby, 2001 ▶); data reduction: *X’pert Data Collector*; method used to solve structure: coordinates taken from an isotypic compound (Thangadurai *et al.*, 2001 ▶); program(s) used to refine structure: *GSAS* and *EXPGUI*; molecular graphics: *VESTA* (Momma & Izumi, 2008 ▶); software used to prepare material for publication: *publCIF* (Westrip, 2009 ▶).

## Supplementary Material

Crystal structure: contains datablocks I, global. DOI: 10.1107/S1600536809018157/br2107sup1.cif

Rietveld powder data: contains datablocks I. DOI: 10.1107/S1600536809018157/br2107Isup2.rtv

Additional supplementary materials:  crystallographic information; 3D view; checkCIF report

## Figures and Tables

**Fig. 1.**
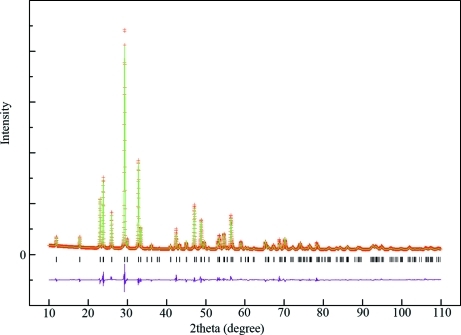
Rietveld difference plot for the multi-phase refinement of RbCa2Nb3O10. The red crosses, and green and pink lines show respectively the observed, calculated and difference plots. Calculated Bragg reflection positions are indicated by black lines for the RbCa2Nb3O10 phase.

**Fig. 2.**
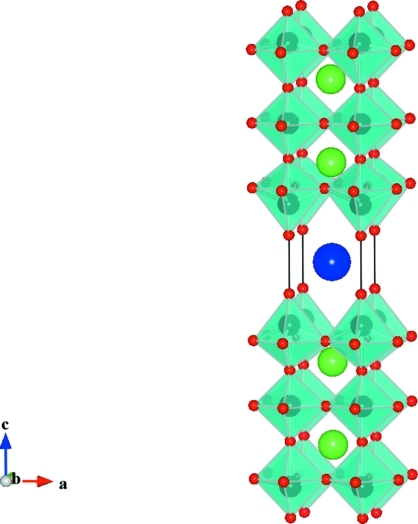
The crystal structure of RbCa2Nb3O10. blue octahedron show NbO3 units with Nb5+ cations as black spheres and O2- anions as red spheres. Large green spheres represent Ca2+ cations and large blue spheres Rb+ cations.

## supplementary crystallographic information

### Comment

The Dion-Jacobson phase which was first discovered by Dion *et al.* (1981), has a general formula A'[A_n-1_B_n_O_3n+1_], where A'is a monovalent ion, A is a divalent alkaline earth metal ion and B is a tetravalent or pentavalent transition metal ion. Related crystal structures of three-layer Dion-Jacobson phase have been reported for KCa_2_Nb_3_O_10_ (Dion *et al.*, 1984), RbSr_2_Nb_3_O_10_ (Thangadurai *et al.*, 2001), and KCa_2_Nb_3_O_10_ (Fukuoka *et al.*, 2000). Although the three-layer Dion-Jacobson phase RbCa_2_Nb_3_O_10_ was first synthesized by Dion *et al.* (1981), its crystal structure has not yet been reported. The structure of RbCa_2_Nb_3_O_10_ has now been refined by the Rietveld method from powder diffraction data in the present communication.

The observed, calculated and intensities difference plots of the Rietveld refinement are shown in Fig. 1. There are some *00 l* preferential orientation which were often observed in the Rietveld refinement of the layered perovskites. Then we applied the March-Dollase option for a correction in the EXPGUI program and obtain the best result finally.

The structure of the compound is illustrated in Fig. 2. The structure consists of three layers of corner-sharing NbO_6_ octahedra that run perpendicular to the *c* axis; adjacent sets of layers are staggered.

Table 1 shows refined interatomic distances and angles for the RbCa_2_Nb_3_O_10_ structure. The octahedra forming the inner layer are less distorted with Nb—O distances ranging from 1.876 (7) to 1.92932 (3)Å (Table 2), which is typical for layered perovskites involving Nb(V). As it is well known in layered perovskites, the NbO6 octahedra forming the outer layer of the slabs are characterized by off-centering of the Nb atoms, leading to four equal equatorial Nb—O distances within the perovskite layers [1.9663 (11) Å], a short Nb—O bond toward the interlayer spacing [1.650 (8) Å], and a long opposite Nb—O bond [2.379 (7) Å]. Such a distortion is quite similar to that encountered in homologous niobates and tantalates where the niobium shows an out-of-plane distortion, moving away from the more positively charged calcium towards the rubidium layer. Similar behavior has been observed in a number of d^0^ systems containing niobium, tantalum, and titanium. This has been attributed to a second-order Jahn-Teller effect. Concerning the interlayer, the rubidium ions are coordinated with eight terminal oxygen atoms to form the same eight Rb—O bonds [3.318 (4) Å]. These distances, as for the Ca—O bonds [2.560 (4)–2.9207 (22) Å] are close to those commonly observed in layered perovskites.

### Experimental

RbCa_2_Nb_3_O_10_ powders were prepared by a conventional solid state reaction described previously (Byeon *et al.*, 2003). All starting materials were of analytical grade and were used without further purification. Stoichiometric amounts of CaCO_3_ and Nb_2_O_5_ with a 50% molar excess of Rb_2_CO_3_ were mixed together and heated in air at 1423 K for 24 h (heating rate 5 K /min). The calcination procedure was repeated one time after grinding to ensure a complete reaction. A 50% molar excess of Rb_2_CO_3_ was used in the reaction to offset the volatilization of the alkali oxides at the synthesis temperature. The products were washed thoroughly with distilled water to remove excess alkali oxides, and were then dried at 393 K overnight.

### Refinement

All peaks of the XRD pattern could be indexed on a tetragonal cell and the systematic absences show simple tetragonal symmetry. The P4/*mmm* crystal structure of RbSr_2_Nb_3_O_10_ (Thangadurai *et al.*, 2001) was used as a starting model for the Rietveld refinement of the structure of RbCa_2_Nb_3_O_10_. The corresponding isotropic atomic displacement parameters of all oxygen atoms are constrained to be equal. The March-Dollase option in the EXPGUI program was applied to correct *00 l* preferential orientation which were often observed in the Rietveld refinement of the layered perovskites.

### Crystal data

**Table:**

RbCa_2_Nb_3_O_10_	*D*_x_ = 4.520 Mg m^−^^3^
*M**_r_* = 604.34	Cu *K*α radiation, λ = 1.54178 Å
Tetragonal, *P*4/*m**m**m*	*T* = 298 K
Hall symbol: -P 4 2	Particle morphology: plate-like
*a* = 3.85865 (6) Å	white
*c* = 14.9108 (3) Å	flat sheet, 10 × 15 mm
*V* = 222.01 (1) Å^3^	Specimen preparation: Prepared at 1423 K
*Z* = 1	

### Data collection

**Table:**

PANalytical X'pert PRO diffractometer	Data collection mode: reflection
Radiation source: sealed tube	Scan method: continuous
graphite	2θ_min_ = 10.01°, 2θ_max_ = 109.99°, 2θ_step_ = 0.02°
Specimen mounting: packed powder pellet	

### Refinement

**Table:**

Refinement on *F*^2^	? data points
Least-squares matrix: full	Profile function: pseudo-Voigt
*R*_p_ = 0.035	26 parameters
*R*_wp_ = 0.053	0 restraints
*R*_exp_ = 0.008	*w* = 1/[σ^2^(*F*_o_^2^) + (0.0677*P*)^2^] where *P* = (*F*_o_^2^ + 2*F*_c_^2^)/3
*R*(*F*^2^) = 0.08530	(Δ/σ)_max_ = 0.020
χ^2^ = 6.452	Preferred orientation correction: March–Dollase (Dollase, 1986) AXIS 1 Ratio= 0.95964, h = k = 0, l = 1. Prefered orientation correction range: min = 0.94007, Max = 1.13156

### Fractional atomic coordinates and isotropic or equivalent isotropic displacement parameters (Å^2^)

**Table:**

	*x*	*y*	*z*	*U*_iso_*/*U*_eq_	
Rb1	0.5	0.5	0.5	0.0433 (8)*	
Ca1	0.5	0.5	0.14706 (19)	0.0281 (8)*	
Nb1	0.0	0.0	0.0	0.0127 (6)*	
Nb2	0.0	0.0	0.28537 (8)	0.0134 (5)*	
O1	0.0	0.5	0.0	0.0716 (14)*	
O2	0.0	0.0	0.1258 (5)	0.0716 (14)*	
O3	0.0	0.5	0.2599 (4)	0.0716 (14)*	
O4	0.0	0.0	0.3960 (6)	0.0716 (14)*	

### Geometric parameters (Å, °)

**Table:**

Rb1—O4	3.138 (4)	Ca1—O3	2.560 (4)
Rb1—O4^i^	3.138 (4)	Ca1—O3^ii^	2.560 (4)
Rb1—O4^ii^	3.138 (4)	Ca1—O3^viii^	2.560 (4)
Rb1—O4^iii^	3.138 (4)	Ca1—O3^ix^	2.560 (4)
Rb1—O4^iv^	3.138 (4)	Nb1—O1^x^	1.929320 (30)
Rb1—O4^v^	3.138 (4)	Nb1—O1	1.929320 (30)
Rb1—O4^vi^	3.138 (4)	Nb1—O1^xi^	1.929320 (30)
Rb1—O4^vii^	3.138 (4)	Nb1—O1^viii^	1.929320 (30)
Ca1—O1	2.9207 (22)	Nb1—O2	1.877 (7)
Ca1—O1^ii^	2.9207 (22)	Nb1—O2^xii^	1.877 (7)
Ca1—O1^viii^	2.9207 (22)	Nb2—O2	2.379 (7)
Ca1—O1^ix^	2.9207 (22)	Nb2—O3^x^	1.9663 (11)
Ca1—O2	2.7468 (9)	Nb2—O3	1.9663 (11)
Ca1—O2^i^	2.7468 (9)	Nb2—O3^xi^	1.9663 (11)
Ca1—O2^ii^	2.7468 (9)	Nb2—O3^viii^	1.9663 (11)
Ca1—O2^iii^	2.7468 (9)	Nb2—O4	1.650 (8)
			
O1^x^—Nb1—O1	180.0	O1^viii^—Nb1—O2^xii^	90.0
O1^x^—Nb1—O1^xi^	90.0	O2—Nb1—O2^xii^	180.0
O1^x^—Nb1—O1^viii^	90.0	O3^x^—Nb2—O3	157.75 (32)
O1^x^—Nb1—O2	90.0	O3^x^—Nb2—O3^xi^	87.87 (6)
O1^x^—Nb1—O2^xii^	90.0	O3^x^—Nb2—O3^viii^	87.87 (6)
O1—Nb1—O1^xi^	90.0	O3^x^—Nb2—O4	101.12 (16)
O1—Nb1—O1^viii^	90.0	O3—Nb2—O3^xi^	87.87 (6)
O1—Nb1—O2	90.0	O3—Nb2—O3^viii^	87.87 (6)
O1—Nb1—O2^xii^	90.0	O3—Nb2—O4	101.12 (16)
O1^xi^—Nb1—O1^viii^	180.0	O3^xi^—Nb2—O3^viii^	157.75 (32)
O1^xi^—Nb1—O2	90.0	O3^xi^—Nb2—O4	101.12 (16)
O1^xi^—Nb1—O2^xii^	90.0	O3^viii^—Nb2—O4	101.12 (16)
O1^viii^—Nb1—O2	90.0		

Symmetry codes:  (i) *x*, *y*+1, *z*; (ii) *x*+1, *y*, *z*; (iii) *x*+1, *y*+1, *z*; (iv) ; (v) ; (vi) ; (vii) ; (viii) −*y*+1, *x*, *z*; (ix) −*y*+1, *x*+1, *z*; (x) *x*, *y*−1, *z*; (xi) −*y*, *x*, *z*; (xii) .
